# Development and Feasibility of a Virtual Nutrition Education Intervention for Adults with Non-Dialysis Chronic Kidney Disease

**DOI:** 10.3390/nu18183096

**Published:** 2026-09-21

**Authors:** María F. Santos-López, Pilar C. Castro-Mata, Cyntia C. García-Ramírez, Lucia Pérez-Galván, Astrid Campos-Medina, Ari Cisneros-Hernández, Karla Nuño, Hugo E. Chávez-Chávez, Laura M. Diaz-Canchola, Alfonso M. Cueto-Manzano, Fabiola Martín-del-Campo

**Affiliations:** 1 Unidad de Investigación Médica en Enfermedades Renales, Hospital de Especialidades CMNO, Instituto Mexicano del Seguro Social, Guadalajara 44320, Mexico; fs.fernanda982@gmail.com (M.F.S.-L.); pilar.castro3712@alumnos.udg.mx (P.C.C.-M.); carolinagr9898@gmail.com (C.C.G.-R.); nutlucia.pg08@gmail.com (L.P.-G.); astridcamposmedina@gmail.com (A.C.-M.); aricisneroshernandez@gmail.com (A.C.-H.); a_cueto_manzano@hotmail.com (A.M.C.-M.); 2 Departamento de Educación, Psicología y Salud, Instituto Tecnológico y de Estudios Superiores de Occidente, ITESO, Tlaquepaque 45604, Mexico; karlanuno@iteso.mx; 3 División de Medicina Interna, Hospital General Regional 180, Instituto Mexicano del Seguro Social, Tlajomulco 45653, Mexico; hugo.chavez8965@academicos.udg.mx (H.E.C.-C.); dralauramargaritadiazcanchola@gmail.com (L.M.D.-C.)

**Keywords:** education, nutrition, chronic kidney disease, healthy diet

## Abstract

Background/Objectives: Adherence to a healthy diet has been shown to decrease the incidence and progression of chronic kidney disease (CKD). However, traditional nutrition education for CKD may not always adequately address healthy eating while incorporating patients’ individual needs. The primary objective of this study was to systematically develop a nutrition education intervention for adults with stage 3–4 non-dialysis CKD; the secondarily objective was to evaluate the feasibility of the developed intervention. Methods: The educational material was systematically developed using ADDIE as the methodological framework and the COM-B (Capability, Opportunity, Motivation, and Behavior) model as theoretical reference to promote behavioral change in patients with CKD. Following the theoretical design, renal nutrition experts created the educational scripts, professionally produced video clips, and supporting brochures. The material was delivered digitally to patients with CKD to evaluate the feasibility of the intervention. Results: A virtual educational intervention was developed to increase access for patients with limited mobility or living in rural areas and to enhance practical skills and stimulate interaction through structured exposition. It consists of 4 sessions, including 10 short videos to enhance attention and comprehension of the topics, and 6 digital handouts to reinforce the content. A proportion of 87% of invited patients completed the intervention and the acceptability surveys; in general, the acceptability of the educational intervention was high, with a mean score of 32 out of 35 points. Conclusions: This educational intervention proposes a feasible structured intervention to improve adherence to a healthy diet, which may contribute to the strengthening of nutritional practice in CKD by promoting a patient-centered approach.

## 1. Introduction

Chronic kidney disease (CKD) represents a major public health challenge due to its high prevalence, progressive nature, and associated mortality. CKD is estimated to affect approximately 10% of the global population, making it a condition of considerable importance for healthcare systems worldwide [1]. In this context, strategies to slow disease progression and prevent its complications have become increasingly important. Among these, nutrition therapy constitutes a fundamental component of CKD management, as an appropriate diet can help control metabolic disturbances, reduce kidney workload, improve nutritional status, and enhance patients’ quality of life [2]. However, the complexity of dietary recommendations, together with barriers related to knowledge, motivation, and access to understandable information, hinders adherence to nutritional recommendations [3].

Adherence to dietary recommendations in CKD is influenced by multiple interacting factors that extend beyond nutritional knowledge. Patients frequently report difficulties understanding complex dietary restrictions, receive conflicting nutritional information, experience emotional distress, have limited family support, face financial constraints, and maintain food preferences and cultural eating habits that hinder dietary changes [4]. In addition, they often express uncertainty regarding which foods are appropriate for their condition. Consequently, nutrition education based solely on the delivery of information is often insufficient to promote sustained changes in dietary behavior because it does not adequately address the determinants that influence everyday food-related decisions [5].

Among the dietary changes increasingly recommended for individuals with non-dialysis CKD is a greater emphasis on minimally processed plant-based foods [6,7]. However, patients frequently express concerns regarding the safety of fruits, vegetables, legumes, and other naturally potassium-containing foods, illustrating how misconceptions and insufficient guidance can hinder the implementation of current dietary recommendations [2]. These challenges reinforce the need for educational strategies that address not only knowledge but also the behavioral and contextual determinants of food choices.

Evidence-based educational materials may support self-care and sustainable dietary change in individuals with CKD [8]. However, existing resources are not always developed based on patients’ identified needs, nor are they grounded in behavior-change theories or adapted to their cultural and dietary contexts [9].

The ADDIE method (Analysis, Design, Development, Implementation, and Evaluation) is an instructional recognized framework to design and structure educative programs, applicable to complex learning environments, including multimedia [10]. On the other hand, behavioral science has recognized that interventions designed to modify lifestyle behaviors should be developed using theoretical frameworks capable of identifying the determinants of health-related behaviors [11]. Among these, the COM-B (Capability, Opportunity, Motivation, and Behavior) model proposes that behavior results from the interaction between individuals’ capability to perform an action, the opportunities provided by their physical and social environment, and their motivation to initiate and sustain that action [12]. This model provides a theoretical framework for translating identified barriers and facilitators into targeted intervention components, thereby allowing nutrition education to move beyond the provision of information and address the behavioral and contextual factors that influence everyday food choices [13].

The combination of the ADDIE method for the instructional design and the COM-B model to promote change in eating behavior provides complementary approaches, ensuring that the intervention considers not only pedagogical and technological criteria but also facilitates behavior change. This combination is particularly relevant for patients with CKD [14], where dietary self-management is influenced not only by knowledge but also by skills and motivation.

In addition to theory-informed content, digital delivery may help overcome geographical and logistical barriers to nutrition education [15]. Educational videos, online platforms, and multimedia resources allow patients to review educational content at their own pace, facilitate the involvement of family members or caregivers, and support continued learning outside the clinical setting. These approaches may be particularly valuable in low- and middle-income countries, where access to specialized renal dietitians remains limited and healthcare systems face increasing demands associated with CKD [16]. Nevertheless, evidence describing the systematic development adapted virtual nutrition education interventions for patients with non-dialysis CKD—integrating patient-identified needs, behavior-change theory, and evidence-based dietary recommendations—remains limited.

Therefore, this study aims to systematically develop a virtual nutrition education intervention for adults with stage 3–4 non-dialysis CKD. A secondary objective was to evaluate the feasibility of the developed intervention.

## 2. Materials and Methods

**Figure 1:**
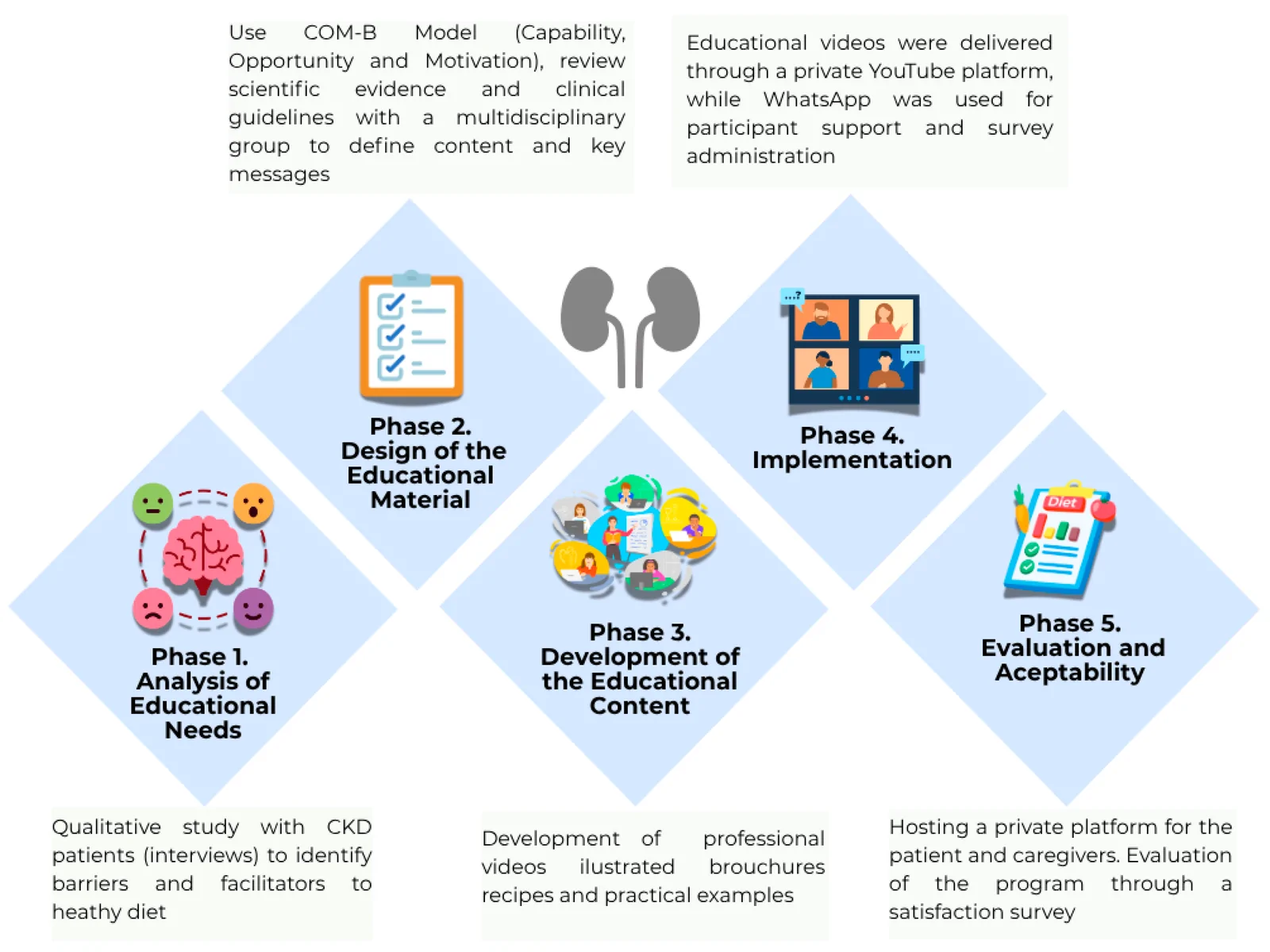
Development process of virtual nutrition educational material-based ADDIE model.

In the present study, the ADDIE method guided the overall development process throughout the analysis, design, development, implementation and evaluation phases, while the COM-B model informed the behavioral content and strategies incorporated within the intervention (Figure 1).

### 2.1. Phase 1. Analysis of Educational Needs

Education needs in the local context of patients were obtained from a previously published qualitative study [3] aimed at exploring the barriers and facilitators related to healthy eating among adults with CKD. In that study, semi-structured interviews were conducted and analyzed using thematic analysis. In addition, evidence from other published studies reporting similar outcomes was reviewed and compared to complement and support the identification of educational needs [5,17]. These studies described barriers including limited knowledge of nutritional recommendations, food preferences, fear of consuming certain foods, emotional responses, financial constraints, lack of family support, and difficulties accessing reliable information. Commonly reported facilitators included motivation to preserve health, support from healthcare professionals, interest in learning, and family involvement. Here, the COM-B model was used to identify factors influencing eating behavior.

### 2.2. Phase 2. Design of the Educational Material

A multidisciplinary team of renal dietitians and nephrologists with expertise in CKD management designed the educational content through an iterative process. During a series of multidisciplinary meetings, the learning objectives and thematic sequence for each session were defined. All educational content was designed according to the most recent recommendations [17] while also emphasizing the potential benefits of a plant-based dietary pattern [13]. Furthermore, all educational content, particularly the recipes, was designed with the context of Mexican cuisine in mind, using local and traditional ingredients that are also the most affordable and accessible for individuals of lower–middle socioeconomic status.

The previously identified barriers and facilitators were also mapped to the COM-B components of capability, opportunity, and motivation. This mapping was subsequently used to review and adjust the learning objectives and develop the educational strategies, addressing factors that limited adherence to a healthy dietary pattern.

The application of the COM-B model in the material was integrated as follows:

- Capability: Focused on increasing participants’ knowledge through the provided educational information and developing practical skills through observation and demonstration of healthy dietary practices;
- Motivation: Addressed participants’ beliefs about the consequences of dietary behaviors, strengthened their confidence in making healthy food choices, and encouraged them to complete educational intervention together with a family member or caregiver;
- Opportunity: Focused on facilitating healthier food choices by providing practical, culturally appropriate recommendations and reinforcing key messages through the accompanying educational brochure.

The educational material incorporated a variety of teaching and learning strategies designed to facilitate understanding, encourage active patient participation, and promote dietary behavior change [18]. The selection of these strategies was based on the principles of health education, adult learning, and behavior change, with the aim of enabling participants not only to acquire knowledge but also to develop the practical skills required to apply nutritional recommendations in their daily lives [10,19].

The following main educational strategies were selected:

Educational presentation. Short educational videos were developed to deliver fundamental concepts related to CKD, healthy eating patterns, and nutrition through structured oral presentations. This teaching approach was designed to facilitate the understanding of specific topics, promote participants’ reflection, and enhance comprehension of complex nutritional concepts.

Demonstration. This strategy consisted of showing participants how to perform specific dietary practices through instructor-led visual demonstrations. It illustrated healthy foods, cooking techniques, and the preparation of simple CKD-appropriate recipes using locally available ingredients. By observing these practical demonstrations, participants were able to better understand how to translate nutritional recommendations into their daily lives and develop confidence in applying them.

Use of Real-Life Examples. This strategy employed everyday situations to contextualize nutritional recommendations and facilitate the understanding of complex concepts. Examples included healthy recipes, plant-based food substitutions, and practical kitchen situations commonly encountered by patients with CKD.

Simulation-based training. This strategy recreated realistic situations that patients commonly encounter in their daily lives, allowing them to mentally rehearse the application of the knowledge and decision-making skills acquired during the educational sessions. Scenarios included choosing healthier food options while grocery shopping or eating away from home, planning balanced meals, and selecting appropriate alternatives to foods high in sodium or phosphorus. The aim was to strengthen participants’ confidence and ability to transfer the acquired knowledge into everyday dietary decisions.

Microlearning. Microlearning is an educational strategy based on short audiovisual learning resources, designed to facilitate the acquisition of knowledge through a structured presentation of information derived from patients’ perceived needs and compared with scientific evidence, incorporating examples from everyday life. This approach promotes self-directed learning and improves the understanding of complex nutritional concepts [20].

Brochures. Digital brochures were proposed as complementary educational resources to reinforce the content presented in the videos, facilitate learning, improve knowledge retention, and support the application of nutritional recommendations within the home environment. These materials also provide a convenient reference for participants and their family members after completing each module [21,22].

### 2.3. Phase 3. Development of the Educational Content

Once the intervention’s theoretical framework was established, the educational design was translated into multimedia educational resources through a structured development process. Based on the learning objectives, teaching strategies, and nutritional content defined during the previous phases, the educational content, instructional structure, and preliminary scripts for each video were developed. Every script was designed to ensure a logical progression of concepts, incorporate practical examples, maintain consistent educational messages throughout the intervention, and facilitate participants’ understanding of nutritional recommendations.

The preliminary scripts were reviewed by a multidisciplinary panel of renal dietitians and nephrologists. During a series of meetings, the content of each educational session was critically evaluated to ensure scientific accuracy, consistency with current nutritional guidelines for CKD, clinical relevance, and alignment with the predefined learning objectives. The suggestions of an expert panel were incorporated iteratively until consensus was reached on the final version of each educational resource.

A series of live-action videos was produced to develop nutritional educational content. This format was carefully designed to ensure consistency in information and verbal communication, with the presence of a healthcare professional strengthening the perception of closeness and credibility of educational content.

For the audiovisual components, an appropriate color palette was selected for use throughout the video editing process, along with the graphic style, font size, animations, copyright-free images, lighting, background music, image transitions, and other visual elements.

During the production planning phase, filming logistics were established, including the recording locations (sets), audiovisual equipment requirements, and technical aspects of video production. The project was carried out in collaboration with a team of professional graphic designers who were contracted to provide professional audiovisual services for the production, direction, and post-production editing of each educational video.

The videos were recorded in different settings: at a green-screen studio for the explanatory videos, at a teaching kitchen that operates in accordance with official hygiene regulations for the cooking and practical demonstration videos for nutrition training, and at the research facilities for videos focused on more specific microlearning approaches.

The videos starred the multidisciplinary team, including renal dietitians, general physicians and nephrologists; all team members signed an image rights consent form authorizing the use of their image for educational purposes. Once all videos were recorded, they were reviewed, and editing and spelling errors in the subtitles were corrected, in addition to adjustments of the sequence, images, and color.

Additional audiovisual material included brochures developed to complement the educational videos for each educational session. Canva Pro (Canva Pty Ltd., Sydney, Australia) was used for their design, following the principles of health literacy and multimedia learning. Clear and accessible language, illustrations, photographs, infographics, and visual elements related to a healthy diet for CKD were incorporated, together with practical examples to reduce cognitive load and improve information retention. All educative materials were developed in the Spanish language.

### 2.4. Phase 4. Implementation

#### Participants

Participants were recruited from the nephrology outpatient clinic of Regional General Hospital No. 180, located in Tlajomulco de Zúñiga, Jalisco, Mexico. Adults (age 18–70 years) diagnosed with non-dialysis stage 3–4 CKD and with access to a personal or familiar mobile phone or tablet with Internet connectivity (either private or public network) were included. Patients with serious illnesses (cancer, AIDS, severe liver and cardiac failure) or serum potassium concentrations >5.5 mEq/L and those receiving private nutritional care during the study period were excluded. All subjects had the same opportunity to participate, independently of their ability to use technological devices for educational purposes; when patients were not proficient in using the applications or devices, family members or primary caregivers were asked to be involved in the process and help them.

Patients who met the eligibility criteria were consecutively invited to participate over a six-month recruitment period.

After signing the informed consent, socio-demographic and medical history data, as well as contact details (phone number), were collected through direct interview. Participants were contacted 1–5 days later to receive instructions and initiate the intervention. Each participant received the intervention for a period of two months.

The educational videos were uploaded to a private YouTube^®^ channel and organized into four playlists corresponding to the four educational sessions of the intervention. Access to the content was provided through private links, allowing only enrolled participants to view the educational materials. Every two weeks, participants received the link corresponding to the scheduled session via WhatsApp^®^. Throughout the implementation, the number of video views was monitored to verify participants’ access to the educational content.

WhatsApp^®^ was also used as a communication channel through which participants could ask questions related to the educational content and interact with the research team. In addition, participants received the educational brochures digitally through the same platform.

Although there is no universally accepted sample size for feasibility or pilot studies, methodological literature supports the use of small samples when the purpose is exploratory rather than hypothesis testing. Julious and Hertzog proposed a rule of thumb of 10–40 participants per group for pilot studies when prior information for determining sample size is unavailable [23,24].

### 2.5. Phase 5. Evaluation of Feasibility of the Intervention

Feasibility was evaluated considering the recruitment process and the retention rate (patients that completed the intervention), as well as the acceptability of the intervention.

For the purposes of this study, acceptability was defined as participants’ perception of the appropriateness and usefulness of the educational intervention after exposure to its content [25]. Acceptability was evaluated using two complimentary surveys—one at the end of each educational session and the other at the end of the complete intervention—as follows:

- At the end of each session, participants completed a digital survey, evaluating session-specific aspects that included understandability, usability, and social support, in addition to providing qualitative feedback on the educational material through the following items: A. clarity of the language used, B. comprehension of the educational content, C. accessibility of the audiovisual materials, D. family involvement during the educational process, and E. suggestions for improving the content. For each survey item (A to C), a five-point Likert scale was used (higher scores indicate more favorable perceptions of the educational material), with a total score for each session ranging from 3 to 15 points; the score was calculated by summing the individual scores of the items. Suggestions for improving the educational material and family involvement were assessed using open-ended questions.
- At the end of the four sessions, participants completed an additional face-to-face survey to evaluate the overall acceptability, including comprehension, perceived usefulness, self-efficacy, and perceived behavioral impact of the intervention through the following items: A. easy understanding of the information, B. acquisition of new knowledge, C. motivation to change eating habits, D. relevance and applicability of the content, E. application of the recommendations, F. increased confidence in making food-related decisions, and G. impact on food choices. This survey was also completed using a five-point Likert scale (higher scores indicate more favorable perceptions of the intervention), with a total score ranging from 7 to 35 points; the score was calculated by summing the individual scores of the items. Participants were also asked two open-ended questions to determine whether they would be willing to participate in similar educational interventions in the future and whether they considered the duration of the educational intervention (2 months) to be adequate.

### 2.6. Statistical Analysis

The acceptability evaluation results were analyzed using descriptive statistics. Continuous variables were summarized as mean ± standard deviation (SD) or median (IQR) according to their distribution, whereas categorical variables were presented as frequencies and percentages. Sociodemographic and clinical variables were analyzed descriptively to characterize the study participants. All statistical analyses were performed using IBM SPSS Statistics 2025.

## 3. Results

### 3.1. Phase 1. Analysis of Educational Needs

Several factors were selected from the reviewed qualitative literature due to their influence on dietary behavior in patients with CKD:

Barriers:

Relation with negative effects in disease control refers to patients’ perceptions of how their dietary choices affect the progression and management of CKD and associated comorbidities. Participants frequently expressed concerns that consuming certain foods could worsen kidney function or increase blood glucose, blood pressure, or serum potassium levels.

Preferences/cravings for other foods describes patients’ preference for foods other than those recommended for CKD management, particularly highly processed foods, salty snacks, sugary beverages, and traditional dishes perceived as unhealthy. These preferences often generated feelings of restriction and represented an important barrier to maintaining long-term adherence to dietary recommendations.

Economy refers to the economic limitations that influenced patients’ food purchasing decisions. Participants reported that the perceived high cost of fruits, vegetables, fresh foods, and other recommended products often restricted their ability to follow dietary recommendations, leading them to choose more affordable but less nutritious alternatives.

Lack of time includes barriers related to limited time available for grocery shopping, meal planning, food preparation, and healthy eating. Participants described work responsibilities, household duties, and daily routines as factors that reduced their ability to prepare healthy meals and consistently follow dietary recommendations.

Bad self-perception encompasses patients’ feelings of discouragement, frustration, lack of confidence, and perceived inability to adopt healthier eating habits. Many participants expressed doubts about their capacity to successfully modify long-established dietary behaviors or maintain dietary changes over time, particularly after previous unsuccessful attempts.

Tasted/flavor/craving refers to the influence of taste and food palatability on dietary decision-making. Participants frequently reported preferring foods high in salt, sugar, or fat because they perceived them as more enjoyable than healthier alternatives. In addition, some patients considered food recommended for CKD to be bland or less appealing, which reduced their motivation to adhere to dietary recommendations.

Facilitators:

Willingness to change refers to patients’ motivation and readiness to modify their eating habits to improve their health and slow the progression of CKD. Participants expressed a positive attitude toward adopting healthier dietary behaviors, particularly when they perceived that these changes could improve their quality of life, preserve kidney function, or delay the need for dialysis. Personal commitment, the desire to remain healthy, and concern for family members were frequently identified as motivating factors for dietary change.

Dietary prescription refers to the perception that having a personalized meal plan facilitated adherence to dietary recommendations. Participants reported that individualized dietary prescriptions provided clear guidance regarding food choices, portion sizes, and meal planning, reducing uncertainty about what they could eat and increasing confidence in following nutritional recommendations. Personalized meal plans were seen as practical tools that helped incorporate healthy eating behaviors into daily routines.

Nutritional guidance/education encompasses the educational support provided by healthcare professionals, particularly renal dietitians, to improve patients’ understanding of dietary recommendations. Participants highlighted that receiving reliable nutritional information, resolving misconceptions, and having the opportunity to ask questions increased their confidence in making healthier food choices. Continuous education and professional guidance were perceived as essential facilitators for improving adherence to dietary recommendations.

Alternative preparations and variety of food refers to the availability of practical alternatives that allowed patients to maintain a varied and enjoyable diet while complying with dietary recommendations. Participants considered that learning new recipes, preparing healthier versions of traditional dishes, and incorporating a wider variety of foods reduced feelings of restriction and increased their willingness to follow dietary recommendations. Participants perceived practical cooking strategies and culturally adapted recipes as useful tools for maintaining long-term dietary adherence.

This process facilitated the organization of the educational content and the selection of teaching resources tailored to patients’ expressed needs.

### 3.2. Phase 2. Design of the Educational Material

Once the main barriers and facilitators to adherence to a healthy diet were identified, each finding was systematically analyzed to determine the knowledge and skills that participants needed to acquire. This process informed the development of specific learning objectives and the selection of appropriate pedagogical strategies and educational resources, ensuring that each component of the intervention directly addressed a previously identified patient need. Learning techniques were integrated with nutritional education strategies. For example, the “Demonstration” technique was implemented through videos showing the selection of healthy versus unhealthy foods; for “Use real-life examples” during grocery shopping, healthier decision-making when eating outside the home; and for “Simulation-based training”, the substitution of foods high in sodium or phosphorus with healthier alternatives (Table 1).

### 3.3. Phase 3. Development of the Educational Content

The educational material was developed through 4 sessions that integrated multidisciplinary collaboration and evidence-based nutritional recommendations. The content was organized in a progressive sequence, presented in plain language, and supported by visual resources to facilitate understanding.

The educational program consisted of four thematic sessions as follows:

Session 1 (3 videos, approx. 12 min. total)

- Benefits of a healthy diet for CKD;
- Strategies for selecting healthy foods.

Session 2 (2 videos, approx. 11 min. total)

- Animal and plant protein sources;
- Practical recipes using legumes and seeds.

Session 3 (2 videos, approx. 13 min. total)

- Preparation of fruits and vegetables;
- Healthy beverage choices.

Session 4 (2 videos, approx. 17 min. total)

- Improving flavor without excess sodium;
- Myths and facts about phosphorus and potassium.

Some of the healthy recipes used as examples included chickpea soup, chickpea ceviche with vegetables, oatmeal and beet pancakes, chili peppers in oil, chia pudding, lentil picadillo, and roasted bell pepper cream soup.

**Figure 2:**
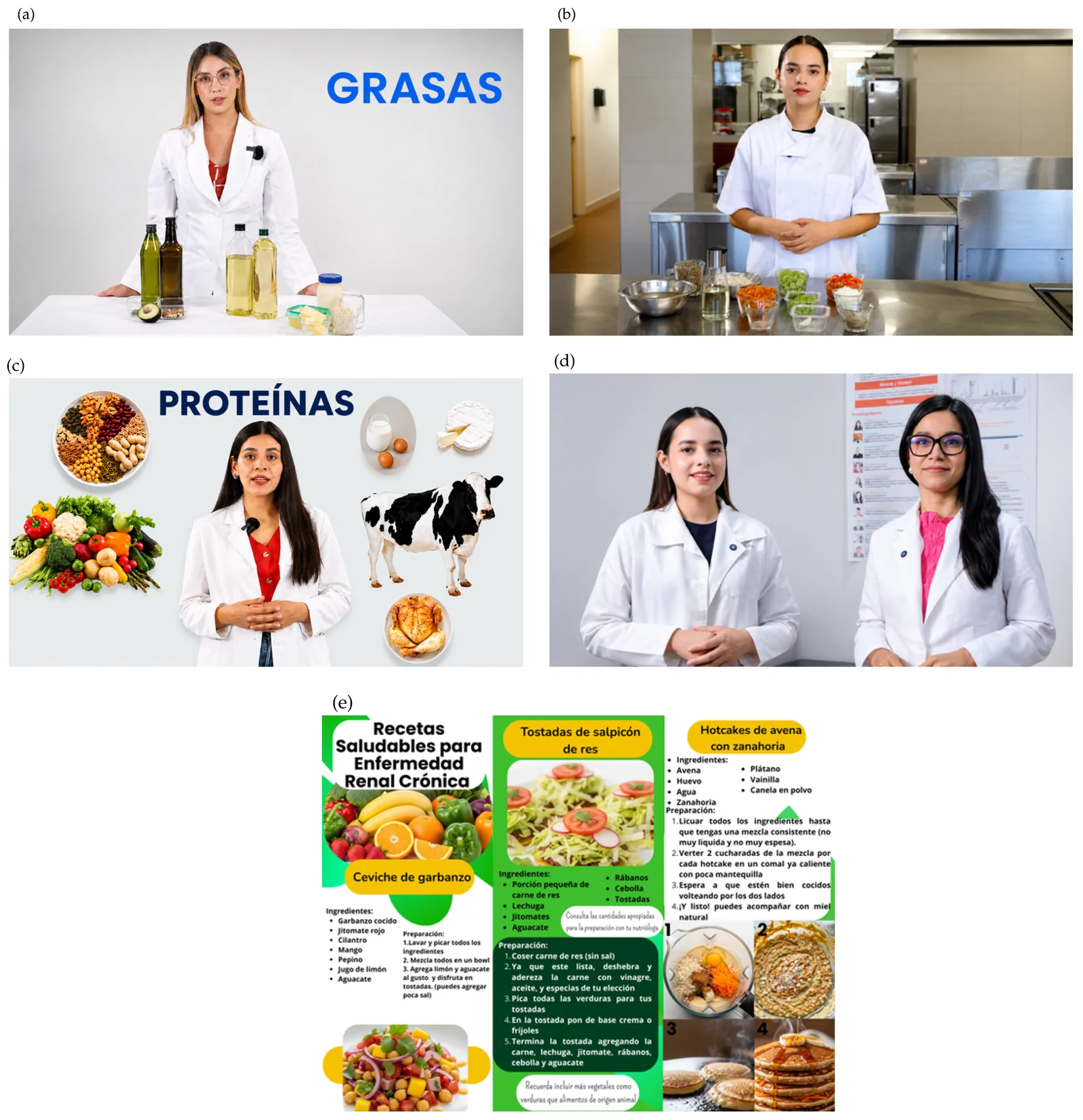
Examples of material education according to the proposed educational strategies. (**a**) Simulation-based training: healthy food selection (dietary fats); (**b**) Use of real-life examples: healthy recipes using legumes; (**c**) Educational presentation: plant-based and animal protein (types of proteins); (**d**) Microlearning: sodium and potassium, myths and facts; (**e**) Brochures: recipes examples (healthy recipes for chronic kidney disease).

The development process resulted in a structured virtual educational intervention comprising audiovisual resources, illustrated educational materials, and learning strategies specifically designed to address the main needs identified among patients with CKD (Figure 2).

### 3.4. Phases 4 and 5. Implementation and Acceptability Evaluation of the Educational Material

Thirty-one patients were eligible and were invited to participate in this study; eight patients refused to participate—five due to personal reasons and only three (9.7%) due to a lack of access to technological devices or Internet connectivity. A total of 23 participants were included in the study. However, contact was lost with three participants who did not respond to messages or telephone calls despite several contact attempts, so they were unable to initiate the intervention. Therefore, 20 participants initiated the education intervention schedule, and all of them completed the sessions and answered the acceptability surveys (retention rate 100%).

Participants had a mean age of 48 years, with women representing most of the sample. Diabetes mellitus was present in two-thirds of the sample, and nearly half of the participants had hypertension; most participants were married or living with a partner and had basic schooling (Table 2).

Table 3 shows participants’ evaluation of each educational session, including the 20 patients that completed the intervention. Overall, participants scored all sessions favorably in terms of comprehension, accessibility of the viewing schedule and clarity of the information.

Regarding family support during the viewing of the educational material, 11 participants (47.8%) reported watching all four educational sessions with a family member, friend, or their children.

Regarding the open-ended question about suggestions for improving the educational materials, participants did not suggest any changes. Instead, they provided comments such as: “It was good,” “Everything seemed good to me”, “I liked the recipes”, and “I liked that the videos were short”.

Results of the acceptability questionnaire administered after completion of all educational sessions are shown in Table 4. Overall, most participants scored the educational material favorably across all evaluated items. The mean overall acceptability score was 32 out of a maximum of 35 points.

All participants reported that they would be willing to participate in future similar nutrition education interventions. Regarding the duration of the intervention (2 months), 57% of participants considered it to be adequate, whereas 43% perceived it as too short.

## 4. Discussion

The main findings of this study demonstrate that it is feasible to develop virtual educational material using a systematic, patient-centered approach to address the barriers that limit adherence to a healthy diet among individuals with CKD. Integrating the findings of previous studies addressing barriers and facilitators for adopting a healthy diet in CKD, along with a behavior change framework, enabled the development of educational materials designed not only to increase knowledge but also to strengthen the practical skills, motivation, and social support necessary to promote sustainable dietary behavior change.

The selection of COM-B was supported by findings from the previous qualitative study, which showed that barriers and facilitators to healthy eating among patients with CKD extended beyond nutritional knowledge and included food preferences, motivation, emotions, time constraints, economic factors, accessibility, and social and family support [3]. These determinants could be conceptually mapped onto the capability, opportunity, and motivation components of COM-B, providing a theoretical basis for translating patient-identified barriers and facilitators into specific educational strategies.

One of the main strengths of the present study is that the educational material was developed through a systematic methodological process (ADDIE) in which the prior identification of barriers and facilitators to adherence to a healthy diet served as the foundation for defining the educational content, teaching strategies, and instructional resources. Although educational interventions targeting patients with CKD are based primarily on clinical practice guidelines and expert knowledge [26,27,28], to the best of our knowledge, this is one of the first studies to describe a development process that integrates previously identified barriers to and facilitators of healthy eating in the target population, allowing the intervention to be adapted to the patients’ dietary context. This patient-centered approach may facilitate the development of more relevant and potentially more effective interventions for the promotion of sustainable behavioral change and provides a methodological framework that could be replicated in different settings.

This finding is consistent with the growing recognition that patient-centered interventions are more relevant and have greater potential to modify health-related behaviors [8]. Previous systematic reviews have shown that educational interventions in CKD can improve patients’ knowledge and support self-management; however, considerable heterogeneity exists in the development and reporting of these interventions, and many educational materials lack characteristics that facilitate behavioral change and patient engagement [15,29].

From a theoretical perspective, our findings support the principles of the COM-B model, which proposes that behavior change occurs when individuals have the capability to perform a behavior, the opportunity to carry it out, and sufficient motivation to sustain it [5]. In the present study, the barriers and facilitators identified through qualitative research allowed these three components to be operationalized during the design of the intervention. For example, knowledge gaps and difficulties in food selection were addressed through strategies aimed at strengthening capability; barriers related to the family environment, food costs, and the availability of healthy food options were considered to enhance opportunity, whereas emotions, beliefs, food preferences, and readiness to change informed the development of strategies designed to increase motivation. These findings suggest that the COM-B model provides a useful framework for translating qualitative evidence into concrete educational components tailored to the context of patients with CKD [30].

The nutritional strategies incorporated into the educational material were based on the most recent scientific evidence regarding the benefits of predominantly plant-based dietary patterns for patients with CKD. Traditionally, dietary recommendations for these patients have focused on restricting foods rich in potassium, phosphorus, and protein, often resulting in highly restrictive dietary patterns that are difficult to maintain over the long term [7]. However, during the past decade, substantial evidence has supported a paradigm shift toward dietary patterns with a greater proportion of plant-based foods, emphasizing fruits, vegetables, legumes, whole grains, and nuts, provided that their implementation is individualized and supervised by healthcare professionals [27].

Several observational studies and reviews have shown that predominantly plant-based dietary patterns are associated with slower CKD progression, reduced cardiovascular risk, improved blood pressure control, and a lower dietary acid load [28]. Although concerns regarding the potassium content of certain plant foods persist, current evidence indicates that, in carefully selected and monitored patients, the potential benefits of increasing plant food consumption outweigh the risks when recommendations are individualized and accompanied by appropriate nutritional education [29].

Consistent with this evidence, the present educational material did not promote generalized dietary restrictions. Instead, it emphasized the selection of plant-based foods, the consumption of plant proteins, and the incorporation of healthy recipes adapted to patients’ preferences and cultural context. This approach is consistent with current recommendations, which recognize predominantly plant-based dietary patterns as a promising nutritional strategy for the comprehensive management of CKD and highlight the importance of providing individualized nutritional education to enable patients to implement these recommendations safely and sustainably [30,31]. An important consideration for implementing nutrition education interventions across different populations is the variability in food availability, dietary patterns, and culinary practices. Although this intervention was based on current CKD recommendations, the foods and recipes reflected the context of the study population; therefore, implementation in other settings may require adaptation according to local food availability and sociocultural characteristics.

The use of virtual education has been shown to enhance learning, improve understanding, and increase patient engagement in educational programs for chronic diseases [14]. In a study conducted among patients with CKD from a low socioeconomic setting, most participants had access to a smartphone and perceived telemedicine as a useful, necessary, and easy-to-use tool for receiving nutritional counseling, despite having a low educational level and limited knowledge of these technologies [32].

Recent studies have reported that, although a wide range of educational materials is available for patients with CKD, many have important limitations in terms of comprehensibility, actionability, and adherence to health literacy principles [13]. In this context, the present program sought to overcome these limitations by using plain language, visual aids, culinary demonstrations, and practical recommendations directly related to the challenges identified by participants.

Finally, the virtual and asynchronous format of the program represents another important strength. The possibility of accessing the content at any time, reviewing the educational videos according to individual needs, and sharing the material with family members or caregivers may facilitate self-directed learning and broaden the reach of educational interventions, particularly for patients who face barriers to attending in-person follow-up visits [33]. Previous studies have reported growing interest in digital educational approaches among individuals with CKD, particularly those that are flexible and personalized to support engagement and self-management [34]. Among the available educational strategies, video-based education has considerable potential because it enables nutritional information to be delivered in a visually engaging and practical manner while allowing patients to access the content according to their individual needs. This approach may be particularly valuable in resource-limited settings and for patients who experience barriers to attending face-to-face consultations [35].

Acceptability is considered one of the key implementation outcomes and reflects users’ perceptions regarding the usefulness and appropriateness of an intervention [9]. The intervention demonstrated high acceptability among the participants included in this study, with a mean score of 32 out of 35 points; however, due to the small sample size, this finding should be interpreted exclusively as a pilot test, and a possible ceiling effect cannot be ruled out. Participants’ favorable ratings regarding the clarity of the content, the usefulness of the educational material, and the audiovisual format suggest that adapting educational resources to previously identified patient needs may enhance their acceptance among end users [36]. Although the present study was not designed to evaluate the educational, behavioral or clinical effectiveness of the intervention, these findings support the feasibility of implementing educational programs developed through participatory, theory-informed approaches and provide a solid foundation for future studies evaluating their effects on dietary adherence and other clinical outcomes [37,38]. The findings should not be considered representative of the broader population of individuals with stage 3–4 CKD, and the generalizability of the acceptability results is limited. The purpose of this assessment was to obtain preliminary information on participants’ perceptions of the developed intervention rather than to estimate population-level acceptability or intervention effectiveness. The limited sample size also precluded meaningful subgroup analyses according to characteristics such as sex, age, CKD stage, or educational level. These variables were collected and are presented only to describe the characteristics of the participants. Future studies should evaluate its effectiveness through randomized clinical trials with medium- and long-term follow-up, incorporating objective measures of dietary adherence, diet quality indices, and nutritional biomarkers to determine the effects of the educational program on dietary behavior and patients’ health status.

The educational program was developed based on the barriers, facilitators, sociocultural characteristics, and sociodemographic context identified among Mexican patients with CKD, with the aim of ensuring the relevance and applicability of the educational content. However, this contextual adaptation may limit the generalizability of the educational material to populations with different cultural characteristics, dietary patterns, languages, or healthcare settings. Therefore, although the methodological framework used to develop the intervention can be replicated in other settings, the educational content and audiovisual resources would likely require cultural and contextual adaptation before implementation in other populations.

## 5. Conclusions

The present study provides a methodological framework for the development of nutrition education interventions for individuals with CKD by integrating scientific evidence with patients’ needs and perspectives. The educational program demonstrated high acceptability, supporting the feasibility of this approach and its potential for implementation across different clinical settings. Future studies should evaluate its effectiveness in improving adherence to a healthy diet and long-term clinical outcomes.

## 6. Patents

An intellectual property registration application for the developed educational material is currently underway.

## Figures and Tables

**Table 1:** Translation of qualitative findings into educational content.

Barrier/Facilitator	Educational Session’s Objectives	Educational Strategies
Food preferences, unhealthy eating habits, and misconceptions about healthy eating	1. Participants will be able to identify healthy and unhealthy eating habits to modify those that require improvement. Additionally, they will learn how to make healthier food choices that are easy to understand and apply in their daily lives.	Simulation-based training; real-life examples; visual explanation of healthy food choices
Lack of nutritional knowledge; fear of consuming plant-based foods	2. Participants will learn different techniques for preparing healthy plant protein dishes using locally available ingredients.	Educational presentation; cooking demonstrations; recipe-based learning; practical examples
Time constraints; limited cooking skills; misconceptions about fruit and vegetable intake	3. Participants will learn how to prepare a variety of healthy dishes easily using locally available, affordable fruits and vegetables, as well as healthy beverages.	Use of real-life examples; meal preparation examples; visual guides; reinforcement through brochures
Dietary restrictions, emotional barriers, food preferences, and social environment	4. Participants will learn strategies to improve the flavor and visual appeal of their meals, enabling them to apply these techniques in their daily cooking while incorporating low salt/sodium alternatives.	Microlearning; myth clarification; problem-solving; practical examples; behavior reinforcement

**Table 2:** Demographic characteristics of the study participants.

Variables	Values N = 23
Sex n (%)	
Female	15 (65)
Male	8 (35)
Age (years)	48 ± 12
Presence of diabetes mellitus (n, %)	15 (65)
Presence of hypertension n (%)	9 (39)
Presence of cardiovascular disease (n, %)	2 (8.7)
Stage CKD n (%)	
3a	5 (22)
3b	11 (48)
4	7 (30)
Duration of CKD (years)	2 (0.6–8)
Schooling n (%)	
Elementary school	3 (13)
Middle school	11 (48)
High school	6 (26)
University	3 (13)
Marital status (n, %)	
Single	9 (39)
Partnered	14 (61)

**Table 3:** Evaluation of each session.

Variable	Session 1	Session 2	Session 3	Session 4
Content comprehension	4.5 (4–5)	5 (4–5)	5 (4–5)	5 (4–5)
Accessibility of the educational materials	5 (3–5)	5 (5–5)	5 (5–5)	5 (5–5)
Clarity	5 (4.2–5)	5 (5–5)	5 (5–5)	5 (5–5)
Total score	13.5 (11–15)	14.1 (12–15)	14.3 (12–15)	14.4 (13–15)

Values are presented as medians (IQR). Items were rated on a five-point Likert scale (1 = very little; 5 = very much), with higher scores indicating a more favorable evaluation of the educational materials. The total score was calculated by summing the three item scores and ranged from 3 to 15 points.

**Table 4:** Final evaluation of the intervention.

Variables	Score
Ease of understanding	5 (4–5)
Acquisition of new knowledge	5 (5–5)
Motivation to change eating habits	4 (4–5)
Relevance and applicability	5 (4.2–5)
Application of the recommendations	5 (4–5)
Increased confidence	5 (4–5)
Impact on food choices	4.5 (4–5)
Total score	32 (26–35)

## Data Availability

The raw data supporting the conclusions of this article will be made available by the authors upon request.

## Abbreviations

The following abbreviations are used in this manuscript:

CKD	Chronic Kidney Disease
COM-B	Capacity, Opportunity, Motivation, and Behavior
ADDIE	Analysis, Design, Development, Implementation, and Evaluation
KDIGO	Kidney Disease: Improving Global Outcomes
KDOQI	Kidney Disease Outcomes Quality Initiative
SD	Standard Deviation
IQR	Interquartile Range
